# School Girls’ Luncheons

**Published:** 1892-05

**Authors:** 


### SCHOOL GIRLS' LUNCHEONS.

The Association of Collegiate Alumnæ of Boston have issued a pamphlet full of statistics of what school girls eat and do not eat in New York and Boston. Out of ninety girls who where questioned one morning in one of the public schools, it was ascertained that twelve had eaten no breakfast. Of these twelve, six had brought no luncheon ; the other six had cake, pie, and pickles, or similar indigestible food. A story is told of four girls, an English, a French, a German, and an American girl, who were taken to a restaurant and invited to call for the food that each would like. The English girl ordered roasted beef; the German girl sauerkraut and brown bread ; the French girl called for *pate de foie gras*, and the American girl, without hesitation, ordered pink ice cream and cake.

The following statistics were gathered from a class of forty girls, varying in age from 16 to 20 :

First morning, nine came without breakfast, two without lunch. Second morning, two came without breakfast, one without lunch. Third morning, one came without breakfast, one without lunch. Fourth morning, three came without breakfast. Fifth morning, three came without breakfast. Sixth morning, two came without breakfast. Seventh morning, three came without breakfast, one without lunch. Eighth morning, four came without breakfast.

And so on for some time it seems that nearly every morning there were from one to five who came to school to do a hard day's work upon an empty stomach. The author says : In an inquiry into the matter of lunches one day, I discovered that two brought pickles, two cake only, one fruit only, three bread only, and one cake and fruit only; the rest brought sensible lunches, and I conclude that the maternal eye watches over the lunch if not over the breakfast. Indeed, in matter of lunches, in many large private schools in New York and other large cities, it is customary to provide a warm lunch at noon for the pupils, so that each girl is obliged to eat at that time. I am told that this custom has spread so extensively that some fashionable schools vie with one another in the elegance of their lunches and the style with which they are served. There may thus be danger of running into the opposite extreme.

There is great need for reform in the matter of hasty meals. Girls study late at night, and ought, very wisely, to be allowed to sleep as long as possible, but this often necessitates a hurried meal in the morning. From inquiries made among the same forty girls, I learned that twenty spend not more than fifteen minutes at table at breakfast, and ten less than ten minutes. Piano practice before breakfast is a custom that cannot be too strongly condemned.

In every case where a girl persists in saying that she cannot eat a breakfast, I make special investigations; I sometimes recommend a little exercise before breakfast, and inquire into the ventilation of the sleeping room. In case of girls who habitually bring no lunch, I insist upon it for the next day, and on every side I have met with the most gratifying obedience from pupils, and hearty coöperation from parents.

